# Improved outcome of pediatric patients with acute megakaryoblastic leukemia in the AML-BFM 04 trial

**DOI:** 10.1007/s00277-015-2383-2

**Published:** 2015-04-28

**Authors:** Jana Schweitzer, Martin Zimmermann, Mareike Rasche, Christine von Neuhoff, Ursula Creutzig, Michael Dworzak, Dirk Reinhardt, Jan-Henning Klusmann

**Affiliations:** Department of Pediatric Hematology and Oncology, Hannover Medical School, Hannover, Germany; Ped. Hematology and Oncology, Pediatrics III, University Hospital Essen, Essen, Germany; St. Anna Children’s Hospital and Children’s Cancer Research Institute, Department of Pediatrics, Medical University of Vienna, Vienna, Austria

**Keywords:** Acute megakaryoblastic leukemia, AMKL, FAB M7, AML-BFM 98, AML-BFM 04

## Abstract

Despite recent advances in the treatment of children with acute megakaryoblastic leukemia (AMKL) using intensified treatment protocols, clear prognostic indicators, and treatment recommendations for this acute myeloid leukemia (AML) subgroup are yet to be defined. Here, we report the outcome of 97 pediatric patients with de novo AMKL (excluding Down syndrome [DS]) enrolled in the prospective multicenter studies AML-BFM 98 and AML-BFM 04 (1998-2014). AMKL occurred in 7.4 % of pediatric AML cases, at younger age (median 1.44 years) and with lower white blood cell count (mean 16.5 × 10^9^/L) as compared to other AML subgroups. With 60 ± 5 %, children with AMKL had a lower 5-year overall survival (5-year OS; vs. 68 ± 1 %, *P*_log rank_ = 0.038). Yet, we achieved an improved 5-year OS in AML-BFM 04 compared to AML-BFM 98 (70 ± 6 % vs. 45 ± 8 %, *P*_log rank_ = 0.041). Allogeneic hematopoietic stem cell transplantation in first remission did not provide a significant survival benefit (5-year OS 70 ± 11 % vs. 63 ± 6 %; *P*_Mantel-Byar_ = 0.85). Cytogenetic data were available for *n* = 78 patients. AMKL patients with gain of chromosome 21 had a superior 5-year OS (80 ± 9 %, *P*_log rank_ = 0.034), whereas translocation *t*(1;22)(p13;q13) was associated with an inferior 5-year event-free survival (38 ± 17 %, *P*_log rank_ = 0.04). However, multivariate analysis showed that treatment response (bone marrow morphology on day 15 and 28) was the only independent prognostic marker (RR = 4.39; 95 % CI, 1.97–9.78). Interestingly, GATA1-mutations were detected in six patients (11 %) without previously known trisomy 21. Thus, AMKL (excluding DS) remains an AML subgroup with inferior outcome. Nevertheless, with intensive therapy regimens, a steep increase in the survival rates was achieved.

**Electronic supplementary material:**

The online version of this article (doi:10.1007/s00277-015-2383-2) contains supplementary material, which is available to authorized users.

## Introduction

Acute megakaryoblastic leukemia (AMKL), M7 according to the French-American-British (FAB) classification [1], is a specific subtype of acute myeloid leukemia (AML) that can be clearly distinguished from other subtypes because of its biological and clinical characteristics. Morphologically, AMKL blasts typically show cytoplasmic blebs, which are immunophenotypically positive for CD41, CD42b, and CD61. Myelofibrosis and manifestation as extramedullary disease are common features of AMKL and often hamper diagnosis.

AMKL occurs predominantly in childhood and particularly in children with Down syndrome (DS) [2–5], accounting for approximately 10 % of pediatric AML cases. In contrast to DS-AMKL (∼80 % survival), non-DS-AMKL is an AML subgroup associated with poor prognosis. For some studies, survival rates of only 14–36 % were reported [5–7], whereas for other studies, survival rates of 50–70 % were reported [6, 8]. Within the AML-BFM studies, the poor outcome of non-DS-AMKL in the AML-BFM 87 trial (5-year event-free survival [5-year EFS] 11 ± 7 %, 5-year overall survival [5-year OS] 21 ± 9 %) could be improved by intensifying the therapy in the AML-BFM 93 and 98 trials (5-year EFS 41 ± 6 %, 5-year OS 49 ± 6 %) [2]. However, despite recent advances in the treatment, the optimal treatment strategy remains controversially discussed. Whereas some study groups treat non-DS-AMKL as very high risk, recommending allogeneic hematopoietic stem cell transplantation (HSCT) during first complete remission (CR1) [5], other study groups obtained superior survival rates with intensive chemotherapy alone, which were not further increased by allogeneic HSCT [8].

Similarly, the prognostic impact of cytogenetically defined subgroups could not be clearly defined, mostly due to the small size of the study cohorts. A limited number of specific genetic abnormalities characterizes non-DS-AMKL. Translocation *t*(1;22), which creates the RBM15-MKL1 (alias: OTT1/MAL) fusion protein, is unique to pediatric AMKL [9, 10]. While the St. Jude AML 02 study proposed *t*(1;22) (*n* = 5) as a good prognostic marker [6], other studies suggested the opposite [9]. Also, the prognostic value of other frequently observed cytogenetic aberrations—such as complex karyotype, trisomy 8, 19, or 21, MLL-rearrangements, loss of chromosome 7 or 7q-, or der(3q)—remains open. Here, we report the clinical, cytogenetic, and therapeutic data of 97 children with non-DS-AMKL, treated uniformly according to the two sequential prospective multicenter studies AML-BFM 98 and AML-BFM 04.

## Patients and methods

### Patients

The AML-BFM 98 and AML-BFM 04 studies were randomized, controlled phase III studies running in 75 centers in Germany, Austria, Switzerland, and the Czech Republic. The AML-BFM 98 study opened in July 1, 1998 and closed in June 30, 2003. Between July 1, 2003 and April 30, 2004, the AML-BFM 98 Interim Study, continuing the best arm of the AML-BFM 98 trial, recruited further patients. The AML-BFM 04 study opened in April 2004 and closed in February 2014. The final protocols were approved by the protocol review committee of the German Cancer Aid (DKH) and by the ethics committee of the University of Münster.

Simultaneously, Germany, Austria, and Switzerland (BFM-Core group) participated in the prospective study on allogeneic HSCT vs. chemotherapy for high-risk (HR) childhood AML in first complete remission (AML CR1 HLA id) on behalf of the European Bone Marrow Transplantation (EBMT) Pediatric Working Party and the International BFM Study Group (I-BFMSG), which was approved by the ethics committee of the University of Tübingen. HR patients and family members were required to undergo HLA typing after the assignment to the risk group. All HR patients with a matched sibling donor were eligible for allogeneic HSCT in first complete remission.

The French-American-British (FAB) classification was used for the initial diagnosis of AML [1]. The diagnoses of the FAB M0 and M7 subtypes required confirmation by immunologic methods [1, 11]. To characterize childhood acute megakaryoblastic leukemia, we reviewed 97 patients, diagnosed with AMKL by the BFM Study Group in the studies AML-BFM 98 (*n* = 37) and AML-BFM 04 (*n* = 60), including patients younger than 18 years with de novo AMKL. Patients with DS, myelosarcoma, secondary AML, or pretreatment of more than 2 weeks were excluded.

### Treatment plan

**Fig. 1:**
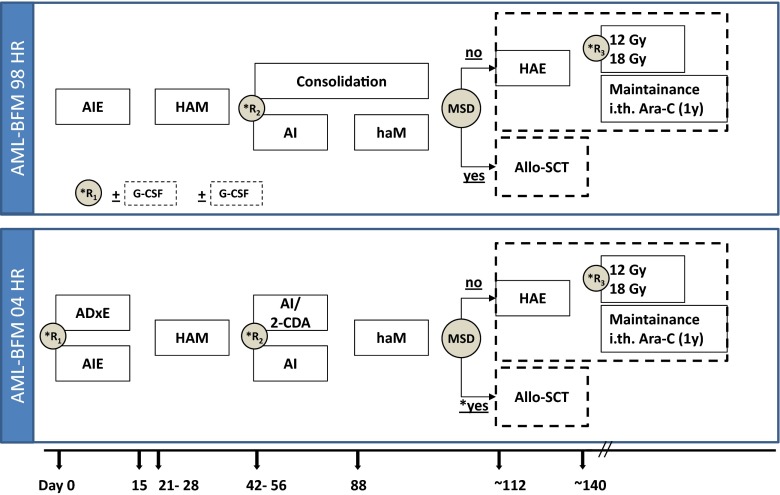
Treatment schedule of AML-BFM 98 and AML-BFM 04 studies for high-risk patients. *AIE* cytarabine/idarubicin/etoposide; *ADxE* cytarabine/l-daunorubicin/etoposide; *HAM* high-dose cytarabine (3 g/m2 q12h over 3 days)/mitoxantrone; *AI* cytarabine/idarubicin; *AI/2-CDA* cytarabine/idarubicin/2-chloro-2-deoxyadenosine; *haM* high-dose cytarabine (1 g/m2 q12h over 3 days)/mitoxantrone; *consolidation* 6-thioguanine/prednisone/vincristine/idarubicin/cytarabine/cyclophosphamide; *HAE* high-dose cytarabine (3 g/m2 q12h over 3 days)/ etoposide; *MSD* matched sibling donor; *asterisk* indicates until 2006; CNS irradiation; *maintenance* 12 months thioguanine/cytarabine; *R1* first random assignment; *R2* second random assignment; *R3* third random assignment

Children with non-DS-AMKL were treated according to regimes AML-BFM 98 and AML-BFM 04, being similar for most parts (Fig. 1).

In AML-BFM 98, patients received chemotherapy with an induction of cytarabine (Ara-C), idarubicin, and etoposide (AIE) and thereafter HAM (high-dose cytarabine, mitoxantrone, cytarabine i.th.), followed by a randomized consolidation therapy (i.e., AI [cytarabine, idarubicin, cytarabine i.th.] and haM [high-dose cytarabine, mitoxantrone, cytarabine i.th.] or the BFM-type 6-week consolidation [6-thioguanine, prednisone, vincristine, idarubicin, cytarabine, cyclophosphamide, cytarabine i.th.]).

In AML-BFM 04, patients received a randomized induction therapy of cytarabine (Ara-C), liposomal daunorubicin (L-DNR), and etoposide (ADxE) or AIE [12]. After the second induction with HAM, consolidation therapy was randomized by introducing 2-CDA (2-chloro-2-deoxyadenosine) to AI as intensification [12–14].

Allogeneic HSCT should be performed in CR1 after consolidation according to the AML CR1 HLA id protocol. Children who were not transplanted in CR1 received one course of HAE as an intensification therapy (high-dose cytarabine and etoposide) and 1-year maintenance therapy (12 months; thioguanine, cytarabine, cytarabine i.th.). After 2006, the indication for allogeneic HSCT was restricted to patients with BM blasts >5 % after second induction. Allogeneic HSCT was performed in 23 SCT centers as reported previously [15]. The recommended standard conditioning regimen for allogeneic HSCT in first complete remission was busulfan and cyclophosphamide.

### Cytogenetic and molecular genetic analyses

Cytogenetic analyses were carried out and centrally reviewed in the AML-BFM reference laboratory in Giessen (AML-BFM 98) and Hannover (AML-BFM 04), Germany, as previously described [16]. Comprehensive cytogenetic data from 80 % (*n* = 78) of the included patients (*n* = 97) were available. Complete karyotypes were described according to the International System of Human Cytogenetic Nomenclature [17]. In the AML-BFM 04 study, sequencing of *GATA1* was prospectively performed as previously described [18]. For 15 patients diagnosed before 2004, material was available for retrospective sequencing analysis.

### Statistics

Complete remission (CR) was defined by fulfillment of the Cancer and Leukemia Group B (CALGB) criteria [19], early death (ED) being death before or within the first 6 weeks of treatment. Event-free survival (EFS) was defined as time from diagnosis to the first event. Events were death from any cause, early death, failure to achieve remission, relapse, and secondary malignancy. Failure to achieve remission was considered as an event on day 0. Survival was defined as the time of diagnosis to death from any cause.

The Kaplan-Meier method was used to estimate survival rates [20]. Differences were compared with the 2-sided log-rank test [21]. Standard errors (SEs) were obtained using Greenwoods formula. Cumulative incidence of relapse and death in CR were calculated by the method of Kalbfleisch and Prentice and compared with Gray’s test. The Cox proportional hazards model has been used to obtain the estimates and the 95 % confidence interval of the relative risk for prognostic factors [22]. Differences in the distribution of individual parameters among patient subsets were analyzed using the chi-squared test or Fisher’s exact test for categorized variables and the Mann-Whitney *U* test for continuous variables. The effect of HSCT on survival was tested using the Mantel-Byar method for comparisons of patients treated or not treated with HSCT. For graphic presentation, patients without HSCT and EFS below the median time to transplantation (0.4 years) were excluded. Follow-up was as of June 2014. Computations were performed using SAS (Statistical Analysis System Version 9.3; SAS Institute, Cary, NC).

## Results

### Patient characteristics

Of 1316 pediatric patients with AML enrolled in the population-based studies AML-BFM 98 and AML-BFM 04, 97 (7.4 %) presented with de novo non-DS-AMKL. Patient characteristics are summarized in Table 1. The median age at diagnosis was 1.44 years (range 0–15 years), being notably younger than for other AML subtypes (9.98 years; *P*_Fisher’s_ < 0.0001). The mean white blood cell (WBC) count of children with de novo AMKL (16.5 × 10^9^/L) was lower than of children with other AML subtypes (58.5 × 10^9^/L). Additionally, initial CNS involvement occurred more frequently among other AML subtypes (12.4 %) than among AMKL patients (2.1 %). Patients with AMKL frequently presented with a longer disease history. Only 41.8 % of AMKL patients had an anamnesis less than 3 weeks, as compared to 57.2 % of children with other AML subtypes.

**Table 1:** Patient characteristics

	AMKL (FAB M7)							Other AML subtypes		
	Total		AML-BFM 98		AML-BFM 04			AML-BFM 98 + 04		
	*N*	(%)	*N*	(%)	*N*	(%)	*P* ^a^	*N*	(%)	*P* ^b^
Gender										
Male	53	54.6	21	56.8	32	53.3		625	51.3	
Female	44	45.4	16	43.2	28	46.7	0.83	594	48.7	0.52
Age, years										
<2	64	66.0	24	64.9	40	66.7		241	19.8	
2–5	23	23.7	6	16.2	17	28.3		170	13.9	
≥6	10	10.3	7	18.9	3	5.0	0.06	808	66.3	<0.001
WBC, 10^9^/L										
<20	75	77.3	24	64.9	51	85.0		631	51.8	
>20	22	22.7	13	35.1	9	15.0	0.03	587	48.2	<0.001
CNS involvement										
No	92	94.8	35	100.0	57	96.6		1036	87.6	
Yes	2	2.1	–	–	2	3.4	0.53	147	12.4	<0.001
BM day 15 + 28										
≤5 % of blasts	74	77.9	24	66.7	50	84.7		815	71.1	
>5 % of blasts	21	21.1	12	33.3	9	15.3	0.05	331	28.9	0.16
Anamnesis										
<3 weeks	38	41.8	16	43.2	22	36.7		680	57.2	
≥3 weeks	53	58.2	18	48.6	35	48.3	0.51	508	42.8	0.004
Allo HSCT in 1.CR										
Yes	18	22.8	9	27.3	9	18.8		113	89.5	
No	63	77.8	24	72.7	39	81.3	0.35	962	10.5	<0.001

^a^AML-BFM 98 vs. AML-BFM 04

^b^AMKL vs. other AML subtypes

Comparing AML-BFM 98 and AML-BFM 04, the study population did not significantly differ. We only noted statistical significance in the mean WBC of 19.7 × 10^9^/L vs. 14.5 × 10^9^/L (*P* = 0.002).

### Treatment outcome

**Fig. 2:**
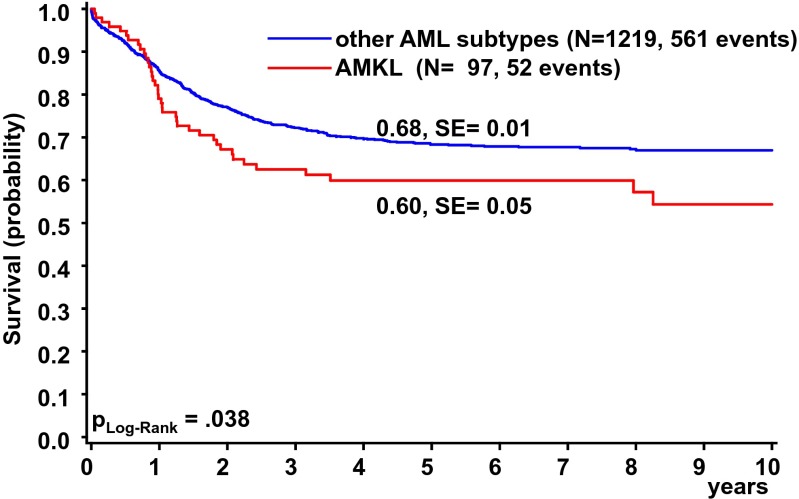
Overall survival of patients diagnosed with non-DS de novo AMKL (*n* = 97) or other AML subtypes (*n* = 1219) in the AML-BFM 98 and AML-BFM 04 studies. Five-year OS is given

With a 5-year OS of 60 ± 5 % (Fig. 2) and 5-year EFS of 47 ± 5 %, children with AMKL revealed a significantly poorer outcome as compared to the total group of children with other AML subtypes (Fig. 2; 5-year EFS 52 ± 1 %, *P*_log rank_ = 0.079). The complete remission (CR) rate of non-DS-AMKL patients was comparable to other children with HR-AML (defined by the reported criteria of the AML-BFM study group [14, 23]). Eighty-one patients (83.6 %) achieved CR, 32 % (*n* = 32) relapsed after first CR, and 4.1 % (*n* = 4) died in first CR. Thirteen patients (13.4 %) were partial- and non-responders (PR; NR) to induction therapy and 3.1 % (*n* = 3) suffered from early death (ED). The cumulative incidence of relapse (CIR) was 45 ± 5 % compared to 39 ± 1 % (*P*_Gray_ = 0.07) for patients with other AML subtypes.

**Fig. 3:**
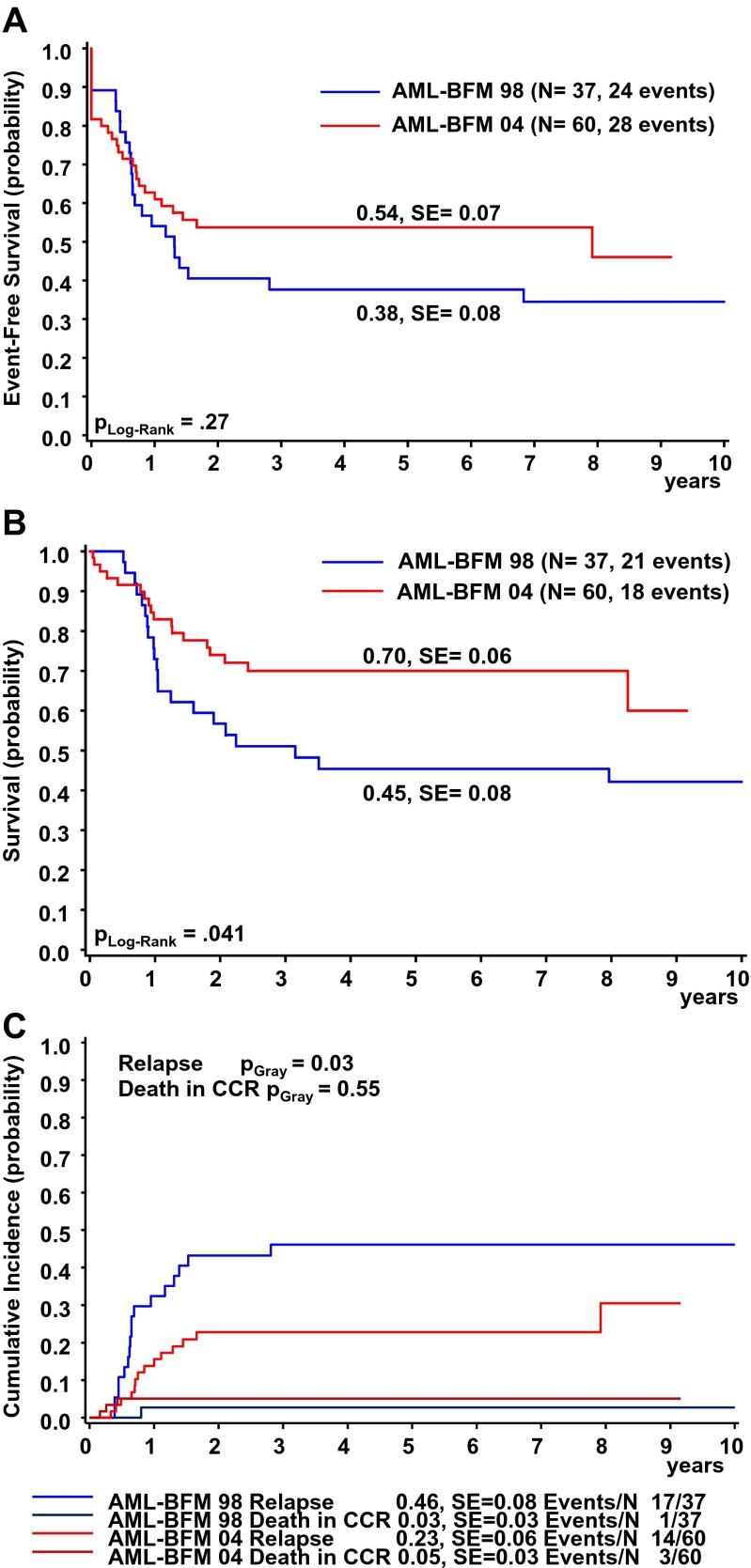
Outcome of AMKL patients in the AML-BFM 98 or AML-BFM 04 studies: **a** Event-free survival. **b** Overall survival. **c** Cumulative incidence of relapse/death. Five-year probabilities are given

Comparing AML-BFM 98 to AML-BFM 04 (Fig. 1), the 5-year EFS increased and the 5-year OS of AMKL patients increased significantly from 45 ± 8 % to 70 ± 6 % (Fig. 3a-b), while CIR decreased from 46 ± 8 % to 23 ± 6 % (Fig. 3c).

**Fig. 4:**
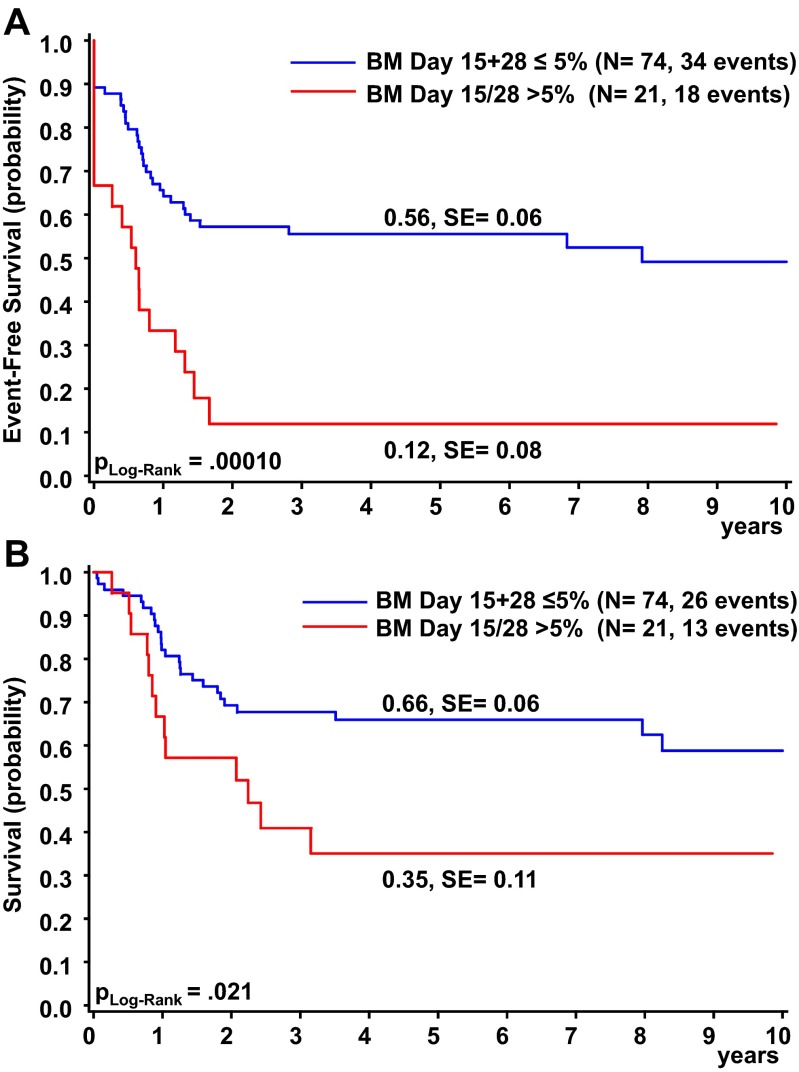
Outcome of AMKL patients based on treatment response: **a** Event-free survival. **b** Overall survival. **c** Cumulative incidence of relapse/death. Five-year probabilities are given. Treatment response was evaluated by bone marrow morphology (>5 % blasts on day 15 or 28)

Although the introduction of liposomal daunorubicin (L-DNR or Dx) in the induction of AML-BFM 04 (randomization of ADxE vs. AIE) resulted in no significant improvement regarding 5-year EFS and 5-year OS (56 ± 12 % vs. 52 ± 8 %, *P*_log rank_ = 0.84; 74 ± 11 % vs. 68 ± 8 %, *P*_log rank_ = 0.57), only 15.3 % of AMKL patients in AML-BFM 04 (ADxE and AIE) displayed a poor treatment response, as assessed by bone marrow morphology on day 15 and day 28 of therapy (>5 % blast cells). This is a clear improvement to AML-BFM 98 with 33.3 % (Table 1). In both cohorts together (AML-BFM 98 and 04), those poor responders had an estimated 5-year OS of only 35 ± 11 % and 5-year EFS of 12 ± 8 %, while good responders (≤5 % BM blasts on day 15 and day 28) showed a 5-year OS of 66 ± 6 % (*P*_log rank_ = 0.021) and 5-year EFS of 56 ± 6 % (*P*_log rank_ = 0.0001) (Fig. 4a, b). The introduction of 2-CDA to the consolidation therapy did not have an impact on the survival rates on AMKL patients.

### Postremission management

**Fig. 5:**
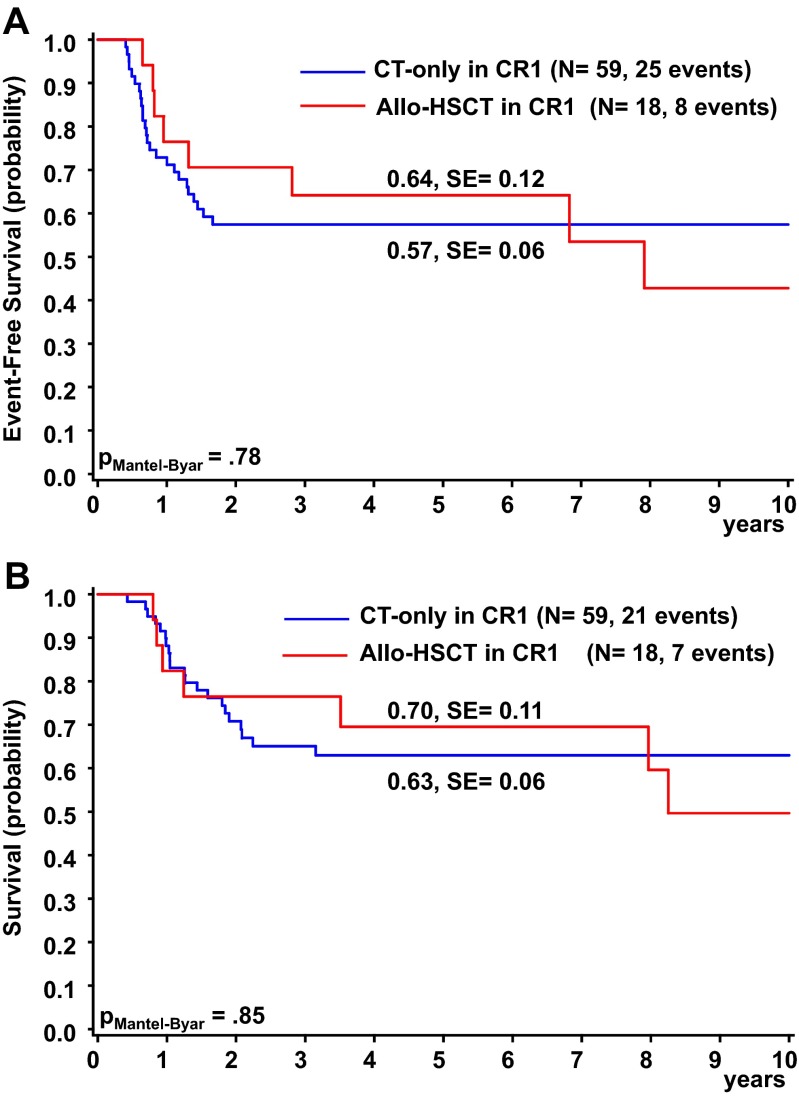
Outcome of AMKL patients assigned to allogeneic HSCT in 1.CR or to chemotherapy only: **a** Event-free survival. **b** Overall survival. Five-year probabilities are given. Patients who did not receive HSCT and had an event before the median time until transplantation (0.4 years) were excluded

Until 2006, allogeneic HSCT in CR1 was recommended for all patients with an HLA compatible sibling donor. To assess the therapeutic effect of allogeneic HSCT compared to chemotherapy alone, we performed as-treated survival analyses taking time to transplant into account. For graphical display patients who did not receive HSCT and had an event before the median time until transplantation (0.4 years) were excluded. In AML-BFM 98, allogeneic HSCT in CR1 was performed on 9 (27 %) non-DS-AMKL patients, whereas 24 patients (73 %) were treated by chemotherapy alone. In AML-BFM 04, the percentages were 19 and 81 %. The outcome was not significantly different in patients undergoing allogeneic HSCT rather than chemotherapy alone in AML-BFM 98 (5-year OS: 56 ± 17 % vs. 49 ± 11 %, *P*_Mantel-Byar_ = 0.95; 5-year EFS 44 ± 17 % vs. 45 ± 11 %, *P*_Mantel-Byar_ = 0.6) and AML-BFM 04 (5-year OS: 88 ± 12 % vs. 72 ± 8 %, *P*_Mantel-Byar_ = 0.83; 5-year EFS: 88 ± 12 % vs. 65 ± 8 %, *P*_Mantel-Byar_ = 0.58)(Supplementary Fig. S1). Likewise, we also observed no benefit from allogeneic HSCT when analyzing the total cohort of AMKL patients (AML-BFM 98 and 04; Fig. 5a, b, Table 2).

**Table 2:** 5-year EFS/OS of defined subgroups in AMKL (AML-BFM 98 + 04)

(*n* = 97)	*N*	Events	EFS (%)	*P*	Deaths	OS (%)	*P*
Gender							
Male	53	28	47 ± 8		21	60 ± 7	
Female	44	24	47 ± 7	0.95	18	60 ± 8	0.92
Age, years							
<2	64	35	46 ± 6		25	60 ± 6	
2–9	23	11	50 ± 11		8	64 ± 10	
≥10	10	6	50 ± 16	0.93	6	48 ± 16	0.36
WBC, 10^9^/L							
<20	75	41	46 ± 6		31	59 ± 6	
≥20	22	11	52 ± 11	0.87	8	65 ± 11	0.99
BM day 15 + 28							
≤5 % of blasts	74	34	56 ± 6		26	66 ± 6	
>5 % of blasts	21	18	12 ± 8	<0.001	13	35 ± 11	0.02
Anamnesis							
<3 weeks	38	15	64 ± 8		13	69 ± 8	
≥3 weeks	53	36	31 ± 7	0.01	25	51 ± 7	0.23
Postremission management							
Allogeneic HSCT in 1.CR	18	8	64 ± 12		7	70 ± 11	
Chemotherapy only	59	25	57 ± 6	0.78	21	63 ± 6	0.85
Cytogenetics							
Normal	13	5	62 ± 13	0.30	5	60 ± 14	0.86
Complex	25	11	55 ± 10	0.60	7	71 ± 9	0.17
*t*(1;22)(p13;q13)	8	6	38 ± 17	0.04	4	63 ± 17	0.30
11q23-aberrations	9	4	51 ± 18	0.93	3	63 ± 17	0.89
der(3)	3	1	67 ± 27	0.50	1	67 ± 27	0.82
+21	21	8	60 ± 11	0.23	4	80 ± 9	0.03
+8	16	8	56 ± 12	0.74	6	68 ± 12	0.62
Monosomy 7	3	2	33 ± 27	0.57	2	33 ± 27	0.19
Hyperdiploid	3	2	33 ± 27	0.46	2	33 ± 27	0.14

### Cytogenetic analyses

Cytogenetic analyses were performed for 78 (80.4 %) children with non-DS-AMKL (Tables 1 and 2). Of note, neither complex karyotype (three or more independent abnormalities including at least one structural abnormality), MLL rearrangement nor monosomy 7/7q- was associated with a significantly worse outcome compared to patients without these aberrations (Table 2, Supplementary Fig. S2A-C). The translocation *t*(1;22)(p13;q13) was found in 8 patients (10.3 %; patient characteristics summarized in Supplementary Table S1), who had a significantly worse 5-year EFS (38 ± 17 %) than patients without this translocation (53 ± 6 %, *P*_log rank_ = 0.039; Table 2 and Supplementary Fig. S2D-F).

Other recurrent aberrations were mainly numerical changes, such as gain of chromosome 8 or 21. Gain of chromosome 21 was associated with better survival rates (5-year OS = 80 ± 9 % vs. 54 ± 7 %, *P*_log rank_ = 0.034; Supplementary Fig. S2G-I).

### *GATA1*-status

Sequencing of the hematopoietic transcription factor *GATA1* was performed on 53 patients. None of these children was previously diagnosed with Down syndrome (constitutional trisomy 21 or trisomy 21 mosaic) or had a history of transient abnormal myelopoiesis (TAM) during the neonatal period. Six patients (11.3 %) were positive for a *GATA1*-mutation, and a gain of chromosome 21 in the leukemic blasts was detected (Table 3). Based on these findings, four patients were diagnosed with trisomy 21 mosaic and two with an acquired trisomy 21. Interestingly, the leukemic blasts of all six patients showed CD7 surface marker expression (Table 3), a common feature of DS-AMKL [24]. Two patients with trisomy 21 mosaic were treated by the reduced intensity ML-DS 2006 treatment recommendations and are in continuous CR without additional therapy. From the remaining four patients, who were treated by standard AML-BFM protocols, one child received matched sibling donor (MSD) HSCT after NR and another one received HSCT after CR2 from a mismatched unrelated donor. Each child with *GATA1*-mutation maintains in continuous CR (Table 3).

**Table 3:** Characteristics of six AMKL patients with *GATA1*-mutation

Patient	Age (years)	Sex	Karyotype	Immunophenotype	Mosaic	Therapy	Outcome
#1	1.2	M	49,XY,der(14)*t*(1;14)(q24 ∼ 25;p11),+13,+21,+21c[14]	CD33/CD34/CD117/CD13/CD36/ CD41/CD42b/CD4^low^/CD7	Yes	ML-DS 2006	CCR
#2	0.7	M	48,XY,+8,+21[4]	CD117/CD33/CD235a/CD61/CD7	No	AML-BFM 04	CCR
#3	1.4	M	48,XY,add(7)(p2?2),+8,del(13)(q1?4),+21c[7]	CD33/pCD34/CD117/CD13/CD36/ pCD42b/CD4^low^/CD7	Yes	ML-DS 2006	CCR
#4	2.4	F	46,XX,del(9)(q?13q33),der(19) *t*(1;19)(q31;p13),der(21;21)(q10;q10)?c	CD33/pCD34/CD117/CD13/CD56/ CD36/pCD42b/CD7	Yes	AML-BFM 04	CCR
#5	1.3	M	47,XY,*t*(3,13)(q?26;q?13 ∼ 14)del(13)(q?14q22),+21c[cp14]	CD33/CD34/CD117/CD56/ CD36/CD41/CD7	Yes	AML-BFM 04	CCR after MSD HSCT in NR
#6	3.2	F	47,XX,*t*(3;7)(q25;p15),+21	CD33/CD34/CD117/CD56/ CD36/CD42b/CD7	No	AML-BFM 98	CCR after MMUD HSCT in CR2

### Multivariate analysis

In the multivariate analysis for 5-year EFS, including sex, age, WBC more than 20 × 10^9^/L, BM blasts more than 5 % on day 15 or 28, as well as cytogenetic subgroups (normal and complex karyotype, *t*(1;22)(p13;q13), gain of chromosome 8 (+ 8), gain of chromosome 21 (+ 21), monosomy 7 and der(3)) as risk factors, only poor treatment response (>5 % blasts after 15 or 28 days; RR = 4.39; 95 % CI, 1.97–9.78; *P*_*x2*_ = 0.0003) was of independent prognostic significance (Table 4). For translocation *t*(1;22)(p13;q13), which was statistically significant in the univariate analysis, multivariate analysis indicated a trend towards poor prognosis (*P*_*x2*_ = 0.07; Table 4).

**Table 4:** Multivariable Cox regression analysis of clinical factors and cytogenetics for EFS

	RR	95 % CI	*P*
Gender	1.0	0.5–2.1	0.96
Age	1.1	0.6–1.9	0.81
WBC >20x10^9^/L	0.9	0.4–2.3	0.90
BM day 15/25, >5 % blasts	4.4	2.0–9.8	0.0003
Cytogenetics			
Normal	0.6	0.2–2.1	0.47
Complex	1.1	0.4–2.9	0.84
*t*(1;22)(p13;q13)	3.9	0.9–16.3	0.07
+8	0.7	0.2–1.8	0.43
+21	0.7	0.3–1.9	0.50
Monosomy 7	0.4	0.1–2.5	0.35
der(3)	0.5	0.0–4.5	0.51

## Discussion

The overall outcome of AMKL (excluding DS) was extremely poor in the early AML-BFM 87 trial (EFS 11 ± 7 %, 5-year OS 21 ± 9 %), which could be significantly improved in the following trials AML-BFM 93 and 98 (5-year EFS 41 ± 6 %, 5-year OS 49 ± 6 %) [2] by introducing idarubicin as well as high-dose cytarabine plus mitoxantrone to the protocol. With a 5-year OS of 70 ± 6 % in AML-BFM 04, we now achieved the highest survival rate for pediatric non-DS-AMKL patients within the BFM studies.

Our study with 97 patients demonstrates that AMKL is nowadays not necessarily associated with a poor outcome, as we and others previously reported (Athale 2001: SJCRH, *n* = 28, 2-year EFS/OS 14/14 %; O’Brien 2013: AML02 (*n* = 26), 3-year EFS/OS 49/54 %; POG 9421 (*n* = 49), 5-year EFS/OS 35/36 %; Barnard 2007: CCG 2891 (*n* = 53), 5-year EFS/OS 23/28 %) [5–7]. Although we confirmed that AMKL constitutes an AML subgroup with inferior outcome compared to the whole group of AML patients, we could achieve a steep increase in the survival rates by continuous development of the AML-BFM therapy protocols. These results support a Japanese study on a cohort of 21 patients with AMKL, reporting 10-year EFS and OS of 57 and 76 %, respectively [8]. Hence, intensification of the already very intensive therapy regimen and better supportive care may result in improved prognosis in AMKL patients.

Interestingly, the improved survival rates of the AML-BFM 04 study could not be attributed to advances in allogeneic HSCT. Thus, we could not confirm previous studies suggesting that survival rates are significantly better after allogeneic HSCT. The St Jude Children’s Research Hospital reported 2-year OS (30 %) after allogeneic HSCT compared to 0 % after chemotherapy alone, stating that allogeneic HSCT during remission offers the best chance of cure [5]. The European Group for Blood and Marrow Transplantation described that the 3-year OS was 82 % in children after allogeneic HSCT. In their study, also DS-AMKL patients were included, which confused the interpretation of the results [25]. In accordance to Hama et al. [8], the survival rates between patients, who were transplanted in CR1 or who received chemotherapy alone, did not differ significantly, resulting in no proven benefit of allogeneic HSCT in CR1 in our series for non-DS-AMKL. Still, because of the small number of AMKL patients with allogeneic HSCT in CR1, the results should be interpreted carefully.

The response to induction therapy is of independent (multivariate analysis) and strong prognostic relevance. Patients with more than 5 % bone marrow blasts on day 15 or 28 of therapy have a poor 5-year OS of only 35 ± 11 %. Still, the number of non-responders could be reduced in AML-BFM 04 (8 %), compared to AML-BFM 98 (17.1 %). PCR- or flow cytometry-based residual disease monitoring may help to distinguish between increased numbers of normal myeloblasts in the regenerating bone marrow after chemotherapy and persistence of malignant blasts to more accurately determine the treatment response in the future [26–28].

Our cytogenetic analyses mark a difference to earlier reports. Carroll et al. [9] and Duchayne et al. [10] described the translocation *t*(1;22)(p13;q13) to be a good prognostic factor. Contrarily, we demonstrated for 8 patients (10.1 % of our cohort) a significantly lower 5-year EFS compared to patients without this translocation. Though this may be attributable to the small number of patients and the high NR rate, our report puts a clear role of *t*(1;22) as a good prognostic indicator into question. Interestingly, neither monosomy 7/7q-, complex karyotype nor 11q23 aberrations indicated a poor prognosis. A good prognostic factor in our study was gain of chromosome 21, which we found in 21.9 % of the patients (Supplementary Fig. 2G-I). This result is represented in children, who carried a *GATA1*-mutation (*n* = 6). Based on the *GATA1*-status, which is strongly associated with DS-AMKL, four of these patients were diagnosed with trisomy 21-mosaicism that was previously unknown. Two patients did not have constitutional trisomy 21, suggesting that trisomy 21 was acquired as an early step during leukemogenesis. All six patients are in continuous remission after treatment, indicating a similar biology and treatment response of AMKL with acquired or constitutional (DS-AMKL) trisomy 21 in conjunction with GATA1s. Therefore, it is crucial to perform *GATA1* mutation analysis on each patient with pediatric AMKL and it may not be advisable that children with *GATA1*-mutations are stratified to the high-risk arm of AML protocols like other AMKL patients. All children with *GATA1s*-mutation showed CD7 surface marker expression, which could serve as a marker to predict the cases with *GATA1s*-mutation [24]. Based on previous experience [29], two patients of our cohort with trisomy 21 mosaic and GATA1 mutation were successfully treated with a reduced intensity regimen for DS-AMKL patients (according to treatment recommendation of the ML-DS 2006 registry). Similar to DS-AMKL, reduced intensity regimens seem efficient in children with trisomy 21 mosaic and overtreatment should be avoided.

Thus, the focus on cytogenetic analyses remains of high prognostic importance. Especially, the role of the newly defined recurrent cytogenetically cryptic NUP98-JARID1A fusion (*t*(11;15)(p15;q35)) and CBFA2T3-GLIS2-fusion (inv(16)) needs to be addressed [30, 31]. For this reason, international collaborative projects on pediatric non-DS-AMKL are essential to study a broad range of chromosomal abnormalities, which might improve statistical analyses and therefore define a better risk stratification [32]. Current efforts from the international BFM study group might address this issue.

In summary, improved intensified chemotherapy and experience of medical staff secure the step-wise and considerable increase of long-term survival for children with non-DS de novo AMKL. We could not prove a clear benefit for allogeneic HSCT in first complete remission for these patients.

## Electronic supplementary material

ESM 1 (PDF 13 kb)

Fig. S1 (PDF 233 kb)

Fig. S2 (PDF 186 kb)

Table S1 (PDF 18 kb)
