# Supplementary material for: Improved outcome of pediatric patients with acute megakaryoblastic leukemia in the AML-BFM 04 trial
Source: Ann Hematol. 2015 Apr 28;94(8):1327–36. doi: 10.1007/s00277-015-2383-2 (PMC4488462; doi:10.1007/s00277-015-2383-2)
Supplement: Supplementary file 1 — (PDF 13 kb) [file 277_2015_2383_MOESM1_ESM.pdf]

Appendix:

Current principal investigators of the Cooperative AML-BFM trials in Germany:

R. Mertens, Kinderklinik RWTH, Aachen; M. Frühwald/A. Gnekow, I. Kinderklinik des KZVA, Augsburg; Th. Rupprecht, Klinikum Bayreuth GmbH; G. Henze/R. Fengler, Charité Campus Virchow-Klinikum, Berlin; L. Schweigerer, Helios Klinikum Berlin-Buch, Berlin; N. Jorch, Ev. Krankenhaus Bielefeld gGmbH, Bielefeld; U. Bode/D. Dilloo, Universitäts-Kinderklinik, Bonn; H.G. Koch/W.Eberl, Städt. Klinikum, Braunschweig; A. Pekrun, Prof.-Hess-Kinderklinik, Bremen; I. Krause/A. Hofmann, Klinikum Chemnitz gGmbH, Chemnitz; E. Holfeld, Carl-Thiem-Klinikum, Cottbus; M. Paulussen/Th. Wiesel, Vestische Kinderklinik, Datteln; D. Schneider/B. Bernbeck, Kinderklinik der Städt. Kliniken, Dortmund; M. Suttorp/ I. Lauterbach, Universitäts-Kinderklinik Carl-Gustav-Carus, Dresden; A. Borkhardt, Universitäts-Kinderklinik, Düsseldorf; A. Sauerbrey, Helios Klinikum Erfurt GmbH, Erfurt; W. Holter/M. Metzler, Universitäts-Kinderklinik, Erlangen; B. Kremens/G. Fleischhack/A. Eggert Universitäts-Kinderklinik, Essen; Th. Klingebiel/Th. Lehrnbecher, Klinikum d. J. W. Goethe-Universität, Frankfurt; C.M. Niemeyer, Universitäts-Kinderklinik Freiburg; A. Reiter/R. Blütters-Sawatzki, Universitäts-Kinderklinik, Gießen; M. Lakomek, Georg-August-Universität, Göttingen; H. Lauffer/T. Bernig/H. Lode/D. Riske, Universitäts-Kinderklinik, Greifswald; D. Körholz/Ch. Kramm, Martin-Luther-Universität Halle-Wittenberg, Halle; R. Schneppenheim/G. Janka/H. Kabisch/S. Rutkowski, Universitätsklinikum Hamburg-Eppendorf, Hamburg; K. Welte/C. Klein/D. Reinhardt, Zentrum f. Kinderheilkunde d. Med. Hochschule, Hannover; A. E. Kulozik, Universitäts-Kinderklinik, Heidelberg; C. Tautz, Gemeinschaftskrankenhaus Herdecke, Herdecke; N. Graf, Universitäts-Kinderklinik, Homburg/Saar; J. Hermann/J. Beck/B. Gruhn, FSU Jena, Klinik f. Kinder- und Jugendmedizin, Jena; J. Kühr/A. Leipold, Städtische Kinderklinik, Karlsruhe; M. Nathrath/M. Rodehüser, Städt. Kinderklinik, Kassel; M. Schrappe/A. Claviez/A. Schrauder, Universitätsklinikum Schleswig-Holstein, Campus Kiel, Kiel; M. Rister/T. Nüsslein, Städt. Klinikum Kemperhof, Koblenz; F. Berthold, Universitäts-Kinderklinik, Köln; W. Sternschulte/A. Prokop, Städt. Kinderkrankenhaus Riehl, Köln; S. Völpel/N.Brauer/T. Imschweiler, Städt. Krankenhäuser, Krefeld; H. Christiansen/U. Bierbach, Universitäts-Kinderklinik, Leipzig; S. Selle, Kinderklinik St. Annastift, Ludwigshafen; P. Bucsky/ M. Lauten, Universitäts-Kinderklinik, Lübeck; P. Vorwerk/U. Kluba, Universitätskinderklinik, Magdeburg; P. Gutjahr/J. Faber, Klinikum d. Joh. Gutenberg-Universität, Mainz; M. Dürken, Universitäts-Kinderklinik, Mannheim; B. Schütz/B. von Zezschwitz, Klinikum d. Philipps-Universität, Marburg; B. Erdlenbruch, Johannes Wesling Klinikum Minden, Minden; I. Schmid/U.Graubner/C. Klein, Kinderklinik und Poliklinik im Dr. v. Haunerschen Kinderspital (Klinikum der Universität München), München; St. Burdach/A. Wawer, Kinder- und Poliklinik des Klinikums rechts der Isar der Technischen Universität München Kinderklinik Schwabing, München; H. Jürgens, Universitäts- Kinderklinik, Münster; W. Scheurlen/O. Moser, Cnopf'sche Kinderklinik, Nürnberg; H. Müller/R. Kolb, Klinikum Oldenburg gGmbH, Oldenburg; O. Peters/S. Corbacioglu, Klinik St. Hedwig, Regensburg; C.F. Classen, Universitäts-Kinderklinik, Rostock; R. Dickerhoff/H. Reinhard, Asklepios Klinik St. Augustin GmbH, St. Augustin; St. Bielack, Olgahospital, Stuttgart; W. Rauh, Krankenanstalt, Mutterhaus der Borromäerinnen e.V., Trier; R. Handgretinger/H. Scheel-Walter, Universitäts-Kinderklinik Tübingen; K.-M. Debatin, Universitäts-Kinderklinik, Ulm; P.-G. Schlegel, Universitäts-Kinderklinik, Würzburg.

Current principal investigators of the Cooperative AML-BFM Trials in Austria:

Ch. Urban, Universitätsklinik für Kinder- und Jugendheilkunde, Graz; B. Meister, Univ.Klinik für Kinder- und Jugendheilkunde, Innsbruck; K. Schmitt, Landes-Frauen- und Kinderklinik, Linz; W. Sperl/N. Jones, Salzburger Universitätsklinikum - Landeskrankenhaus Salzburg; H. Gadner/M. Dworzak, Zentrum für Kinder- u. Jugendheilkunde im St. Anna Kinderspital, Wien.

Current principal investigators of the Cooperative AML-BFM Trials in Switzerland:

R. Angst, Kantonsspital Aarau, Aarau; T. Kuehne, Univ.-Kinderspital Beider Basel, Basel; J. Greiner, Ostschweizerisches Kinderspital, St. Gallen; M. Beck-Popovic, CHUV, Lausanne; P. Brazzola, Ente Ospedaliero Kantonale Bellinzona, Bellinzona; J. Rischewsky, Kinderspital Luzern, Luzern; J.-P. Bourquin, Universitäts-Kinderklinik, Zürich

Principal investigators in the Czech Republic:

J. Sterba, University Hospital Brno, Brno; V. Mihal, University Hospital Olomouc, Olomouc; J. Stary, University Hospital Motol, Prague.
