# Supplementary material for: Improved outcome of pediatric patients with acute megakaryoblastic leukemia in the AML-BFM 04 trial
Source: Ann Hematol. 2015 Apr 28;94(8):1327–36. doi: 10.1007/s00277-015-2383-2 (PMC4488462; doi:10.1007/s00277-015-2383-2)
Supplement: Supplementary file 2 — (PDF 233 kb) [file 277_2015_2383_MOESM2_ESM.pdf]

Supplementary Figures:

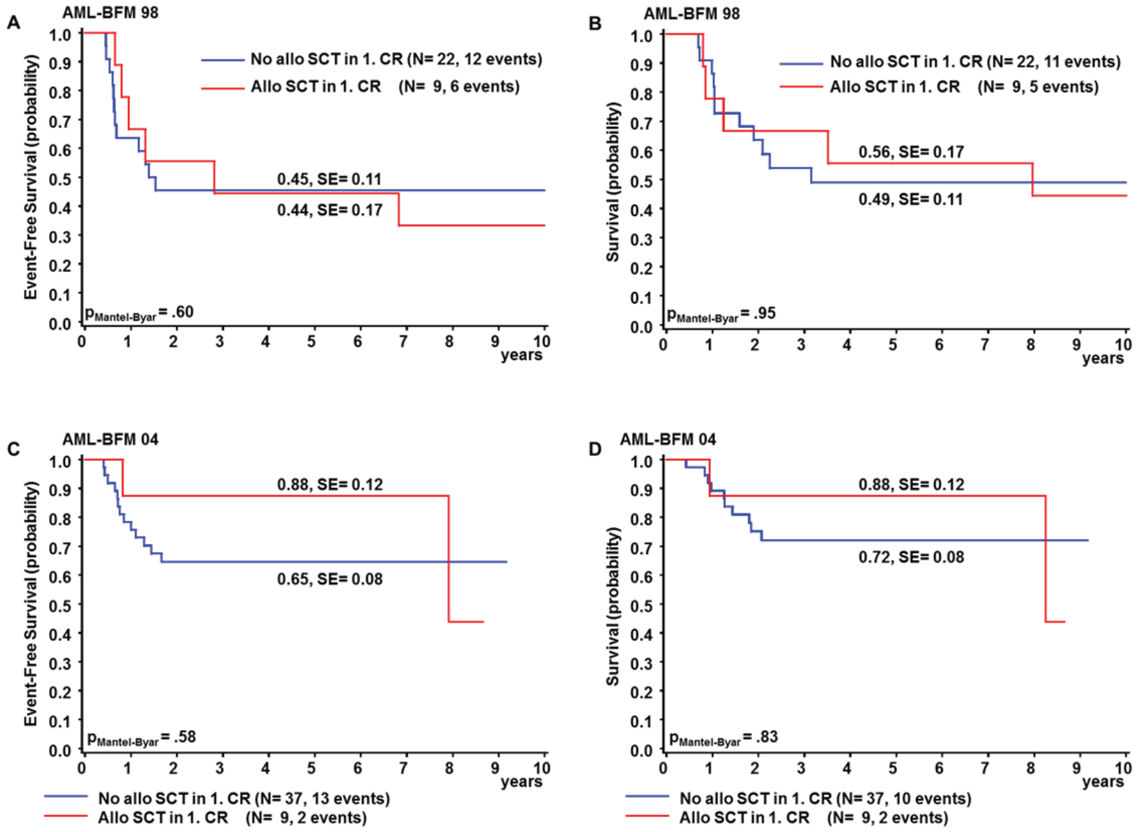

Supplementary Figure S1: Outcome of AMKL patients treated with **allogeneic HSCT in CR1 or to chemotherapy only for the AML-BFM 98 or AML-BFM 04 study.** (A) Event-free survival in AML-BFM 98. (B) Overall survival in AML-BFM 98. (C) Event-free survival in AML-BFM 04. (D) Overall survival in AML-BFM 04. Patients without SCT and EFS below the median time to transplantation (0.4 years) were excluded. 5-year probabilities are given.
