# Supplementary material for: Improved outcome of pediatric patients with acute megakaryoblastic leukemia in the AML-BFM 04 trial
Source: Ann Hematol. 2015 Apr 28;94(8):1327–36. doi: 10.1007/s00277-015-2383-2 (PMC4488462; doi:10.1007/s00277-015-2383-2)
Supplement: Supplementary file 3 — (PDF 186 kb) [file 277_2015_2383_MOESM3_ESM.pdf]

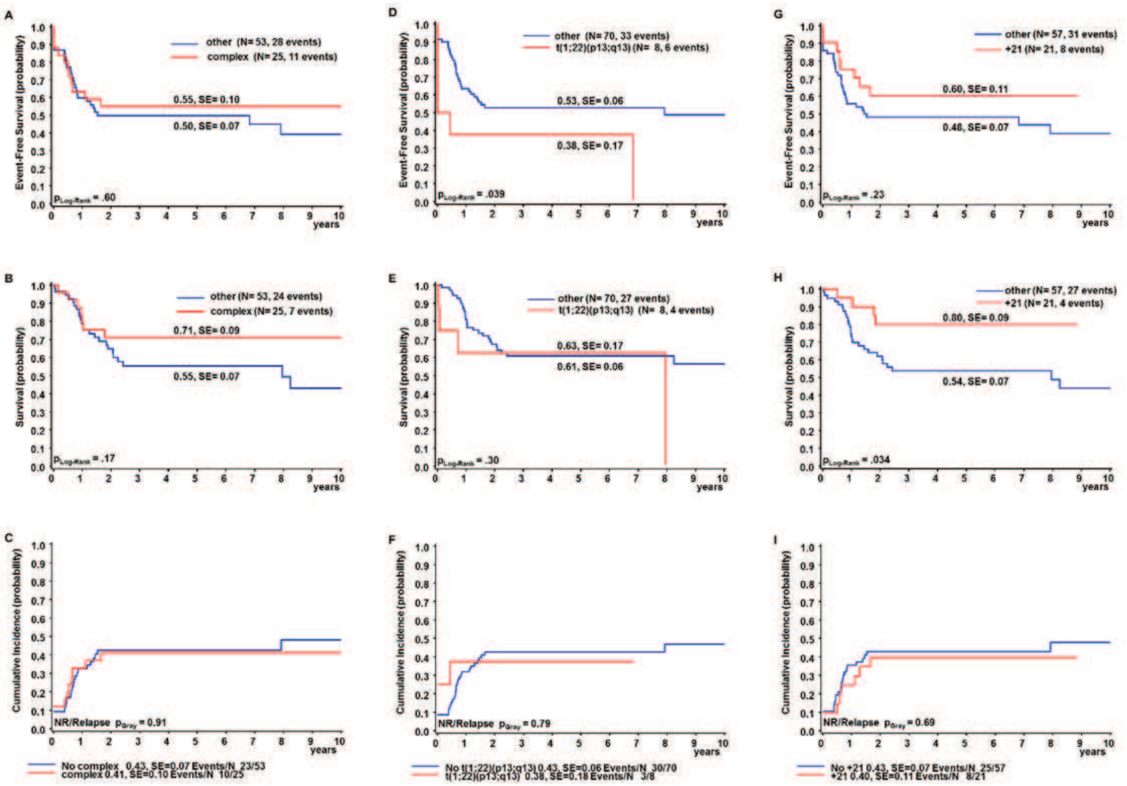

Supplementary Figure S2: Estimated probability of EFS (A, D, G), overall survival (B, E, H) and CIR (C, F, I) for AMKL patients (n=78) based on cytogenetic data: (A-C) Patients with or without complex karyotype. (D-F) Patients with or without t(1;22)(p13;q13). (G-I) Patients with or without gain of chromosome 21. 5-year probabilities are given.
