# Supplementary material for: Improved outcome of pediatric patients with acute megakaryoblastic leukemia in the AML-BFM 04 trial
Source: Ann Hematol. 2015 Apr 28;94(8):1327–36. doi: 10.1007/s00277-015-2383-2 (PMC4488462; doi:10.1007/s00277-015-2383-2)
Supplement: Supplementary file 4 — (PDF 18 kb) [file 277_2015_2383_MOESM4_ESM.pdf]

Supplementary Table:

Supplementary Table S1: Characteristics of eight AMKL patients with t(1;22)(p13;q13)

Patient	Age (y)	Sex	Karyotype	Therapy	Outcome
**#1**	0.12	m	46,XY,t(1;22)(p13;q13)[8]	AML-BFM 98	Death in CCR after MUD HSCT
**#2**	0.24	f	46,XX,t(1;22)(p13;q13)[13]	AML-BFM 04	CCR after MUD HSCT in NR
**#3**	0.59	f	no karyotype, t(1;22)(p13;q13) in FISH	AML-BFM 04	Early Death
**#4**	0.30	f	46,XX,inv(7)(q21q33)[18]; 46,idem,t(1;22)(p31;q12)[2]	AML-BFM 04	CCR after MRD HSCT in NR
**#5**	1.19	m	49,XY,t(1;22)(p13;q13),+2,der(2)(p),+8,+19[cp11]	AML-BFM 04	CR
**#6**	1.52	f	no karyotype, t(1;22)(p13;q13) in FISH	AML-BFM 04	Early Death
**#7**	0.25	m	50~55,t(1;22)(p13;q13),inc	AML-BFM 98	CR
**#8**	6.26	m	68~69,XXYY,der(1)t(1;22)(p13;q13),-1,-2,-4,-4,-5,-5,-6,-7,-9,-9,-11,-12,-12,-13,-14,-15,-15,-16,-17,-18,-18,-20,-20,-22,+mar[cp5]	AML-BFM 98	Death in CCR
